# The Large and Strong Vortex Around the Trunk and Behind the Swimmer is Associated with Great Performance in Underwater Undulatory Swimming

**DOI:** 10.2478/hukin-2022-0087

**Published:** 2022-11-08

**Authors:** Takahiro Tanaka, Satoru Hashizume, Toshiyuki Kurihara, Tadao Isaka

**Affiliations:** 1 Graduate School of Sport and Health Science, Ritsumeikan University, Shiga, Japan.; 2 Faculty of Sport and Health Science, Ritsumeikan University, Shiga, Japan.; 3 Research Organization of Science and Technology, Ritsumeikan University, Shiga, Japan.

**Keywords:** vortex structure, computational fluid dynamics, underwater dolphin kick, swimming performance

## Abstract

Swimmers can produce horizontal body velocity by generating and shedding vortices around their body during underwater undulatory swimming (UUS). It has been hypothesized that the horizontal shedding velocity, area and circulation of the vortex around the swimmer’s body are associated with UUS performance. The purpose of this study was to investigate whether the shedding velocity, area and circulation of vortices around swimmers’ bodies are correlated with the horizontal body velocity during UUS. Computational fluid dynamics (CFD) was conducted to obtain the vortex structure during UUS in nine male swimmers. Morphological and kinematic data of each subject were obtained and used to reconstruct the UUS movement on CFD. The horizontal velocity of the center of vorticity, the area and circulation of vortices around the ventral side of the trunk, dorsal side of shoulder and waist, and behind the swimmer were determined from the simulation results. Positive correlations were found between the vortex area and circulation around the ventral side of the trunk (area r = 0.938, p < 0.05; circulation r = 0.915, p < 0.05) and behind the swimmer (area r = 0.738, p < 0.05; circulation r = -0.680, p < 0.05), and the horizontal body velocity. The horizontal shedding velocity of the center of vorticity of the vortices around the swimmer’s body was not significantly correlated with the horizontal body velocity. These results suggest that the generation of a large and strong vortex around the trunk and behind the swimmer is associated with great UUS performance.

## Introduction

Underwater undulatory swimming (UUS) is an underwater propelling technique used during the start and turn phases of competitive swimming, especially in freestyle, backstroke, and butterfly stroke events. The rule set by Fédération Internationale de Natation (FINA) allows the underwater propelling to be used up to 15 m after the start and each turn (FINA, 2017). Previous studies have reported that the high horizontal body velocity during the start and turn phases is one of the factors for improving the race time (Arellano et al., 1994; Houel et al., 2012; Veiga et al., 2014). UUS at a depth of 0.5 m from the water surface can reduce the wave drag compared to surface swimming (Elipot et al., 2010), and this results in a greater horizontal velocity of the body during UUS (Veiga et al., 2014). Therefore, national-level swimmers use UUS for longer distances (12.2 m) compared with regional level swimmers (10.6 m) (Veiga et al., 2014). These results indicate that UUS performance is an important factor for competitive swimmers.

The vortex phenomena have been investigated to reveal the propulsion mechanism during UUS. The vortices around the ventral side of the trunk, feet, and the dorsal side of the shoulder and the waist are generated and shed during the downward kick (Cohen et al., 2012; Hochstein et al., 2012; Pacholak et al., 2014; von Loebbecke et al., 2009). These vortices around the swimmer’s body are supposed to induce momentum changes in the flow field (Matsuuchi et al., 2009; Takagi et al., 2016; Triantafyllou et al., 2002; von Loebbecke et al., 2009). Hydrodynamically, the change in flow velocity induces changes in the momentum of the flow fields, resulting in increasing the propulsion and/or braking fluid force during UUS (Matsuuchi et al., 2009; Takagi et al., 2016; Triantafyllou et al., 2002; von Loebbecke et al., 2009). Therefore, the increased propulsive fluid force is applied to the swimmer’s body by increasing the vortex velocity, which is shed backward to the swimmers’ body. The vortex area and circulation are also important factors in enhancing fluid force (Imamura and Matsuuchi, 2013). This suggests that swimmers can produce a great horizontal body velocity during UUS by generating a large and/or strong vortex and/or increasing the vortex shedding velocity around the whole body.

Previous studies have evaluated the vortex around the body qualitatively, but not quantitatively. Quantitative studies would reveal which of the vortices around the whole body are important for greater horizontal body velocity during UUS by evaluating the vortex area, circulation and shedding velocity; however, this has not been investigated. The purpose of this study, therefore, was to investigate which of the vortex parameters around the whole body was correlated with horizontal body velocity during UUS. This study will provide important insights for swimmers and coaches to improve UUS performance.

## Methods

The current study quantified the vortex structure during UUS using computational fluid dynamics (CFD). The kinematic and digital swimmers’ model was created for CFD and used to conduct the simulation.

### Participants

Nine male swimmers participated in this study (Table 1). The FINA points were calculated based on participants’ personal records in their specialized styles in long course swimming. The experimental protocol was approved by the local ethics committee of the authors' affiliated university and was conducted in accordance with the guidelines set out in the Declaration of Helsinki. Written informed consent was obtained from each participant prior to the experiment.

**Table 1:** Basic characteristics of study participants.

	The basic data of participants (n = 9)	For verification of validity of the current simulation results	
		One swimmer in the current study	Pacholak et al. (2014)
Age (years)	20.7 ± 2.8	19	Not presented
Body mass (kg)	66.8 ± 4.7	60.9	56.5
Body height (m)	1.69 ± 0.03	1.66	Not presented
Body length (m)	2.25 ± 0.06	2.18	2.20
FINA points	633.6 ± 88.3	690	Not presented
UUS velocity (m/s)	1.41 ± 0.22	1.18	1.18

Abbreviations: UUS - underwater undulatory swimming; FINA - Fédération Internationale de Natation. FINA points represent the competitive level of swimmers.

### Kinematic data collection

Participants performed 15 m UUS with maximum effort using the wall-push start in an indoor pool (7 lanes × 25 m, depth: 1.35 m, water temperature: 30°C). UUS trials were repeated three times at 2 min intervals. Twelve retroreflective markers were attached to the skin over the following bony configurations on the right side of the body: epiphysis of the fifth metatarsal, lateral malleolus, lateral epicondyle of the femur, greater trochanter, iliac horn, lower end of the tenth rib, xiphoid, tragus, acromion, lateral epicondyle of the humerus, styloid process, and tips of the third finger (Atkison et al., 2014; Nakashima, 2009; Yamakawa et al., 2017). Three-dimensional (3D) coordinates of the markers were collected using an underwater motion capture system with eight cameras (Oqus Underwater, Qualysis, Sweden) at a sampling rate of 100 Hz. The capture volume was set between the wall and 15 m from the wall. The error in the dynamic calibration of this system, computed as the standard deviation of the known length of the calibration wand, was 1.1 ± 0.5 mm.

### Collection of 3D swimmers’ model data

**Figure 1:**
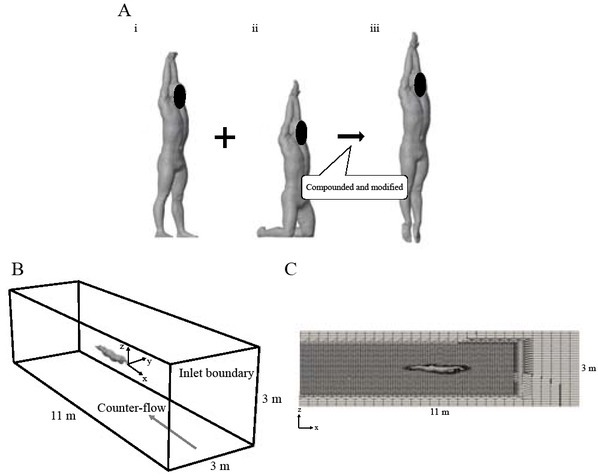
**A**: The swimmers’ model of the standing (i) and the kneeling position (ii) obtained using a 3D laser body scanner, and the glide position (iii) compounded and modified from two models. **B**: Outline of the outer block mesh of the CFD in this study. C: The slice picture of the calculation mesh in this study.

The 3D swimmers’ body data were obtained using a 3D laser body scanner (Body Line Scanner C9036, Hamamatsu Corp., Japan). Participants kept standing and kneeling positions with their arms extended above their heads, wearing white swim caps and shorts during the collection of their body model data (Figure 1Ai, Aii). Due to the limited capture volume of the 3D laser body scanner, 3D swimmers’ body data were collected for the standing and kneeling postures, and then the collected data were compounded to construct the glide position model of the swimmers’ body using 3D animation software (Blender Ver.2.80, Blender Foundation) (Figure 1Aiii). Body length was determined as the length between the tip of the finger and the toe of the swimmers’ model (Table 1).

### Analysis of kinematic data

Kinematic data for the fastest body velocity trial for each participant were chosen for the simulation. The collected data were filtered using a second-order Butterworth low-pass filter with a cut-off frequency of 6 Hz (Yamakawa et al., 2017). This study assumed UUS as a symmetrical movement on the sagittal plane which was defined by the vertical (z-axis) and long (x-axis) axes of the pool lane (Atkison et al., 2014; Higgs et al., 2017). A kick cycle was defined from the highest marker position of the epiphysis of the fifth metatarsal to the next corresponding highest position (Higgs et al., 2017; Yamakawa et al., 2017). The horizontal velocity of the body was computed as the horizontal velocity of the whole-body center of mass on the x-z plane using inertia properties of the body segment for Japanese athletes (Ae et al., 1992). The mean value of this velocity during a kick cycle was then determined. The segment angle was defined as the angle between the long axis of the pool lane and each body segment. These kinematic variables were analyzed for the three kick cycles after the foot passed 7.5 m from the wall (Arellano et al., 2002; Connaboy et al., 2010).

### Reconstruct UUS movement

The joint positions of the whole body were transformed to the coordinate system of the simulation model by the following maneuver. First, the tip of the third finger of the swimmers’ model was located at the origin of the coordinate system of the simulation model (Figure 1B). Then, the vertical displacement of the position of the tip of the third finger was calculated along with the collected data, and the longitudinal and lateral positions were fixed during UUS. Finally, each joint position was geometrically recalculated using the value of the segment length and each segment angle for representation in the coordinate system.

### Computational Fluid Dynamics

The Navier-Stokes equations for an unsteady 3D flow with the turbulence model of the normal k-ε model were solved using OpenFOAM (Ver.6, OpenFOAM Foundation) in this study (Pacholak et al., 2014).


1 ∇⋅u=0

2 $$\frac{\partial u}{\partial t}+(u\cdot \nabla )u=-\frac{1}{\rho }\nabla P+\frac{\eta }{\rho }\Delta u$$

where P is the water pressure, **u** is the fluid velocity vector, ρ is the water density (995.65 kg/m^3^ in this study), and η is the dynamic viscosity of water at time t. The dynamic viscosity of water was calculated as follows:


3 $$\eta =\frac{UL}{Re}$$

where U is the magnitude of the horizontal center of mass velocity, L is the body length of the participants, and Re is the Reynolds number (2.6 × 106) (Pacholak et al., 2014). These equations were solved with the finite volume method with second-order discretization in space and implicit discretization in time using OpenFOAM.

The outer block mesh containing the 3D swimmers’ body model was created with a size of 11 m × 3 m × 3 m (x-y-z) (Figure 1B, C). The outer block mesh of all participants contained 672.4 k ± 3.5 k cells.

Water flowed from the inlet boundary mesh in this study (Figure 1B). The velocity of water was the same as the horizontal center of mass velocity during UUS for each participant. In the first step of the simulation, the water flow and fluid force were simulated during the gliding position for 1 s. In the second step, the simulation during UUS was conducted for the three kick cycles. After every 0.3-0.4 ms, the old surface mesh was updated with a new deformed mesh for preventing the divergence of simulation (Pacholak et al., 2014). The water flow velocity and pressure were interpolated onto the deformed mesh. The time steps were controlled by a Courant number with a maximum value of 0.5.

### Data processing

**Figure 2:**
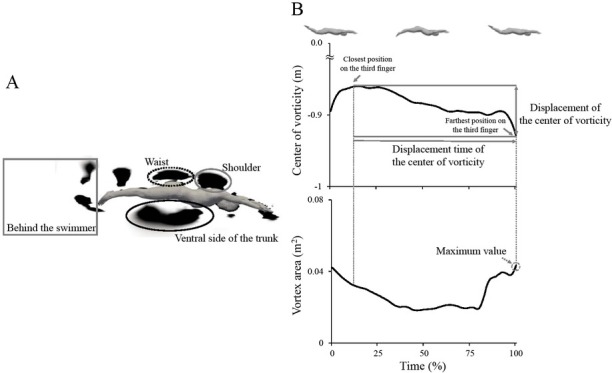
A: The calculation of the vortex area. B: The typical time history data of the center of vorticity (above) and the vortex area (below).


The data processing of CFD was conducted only for the third kick cycle in the current study, since a previous study suggested that the data collected from the first and second kick cycles should be excluded for the data processing of simulation (Pacholak et al., 2014). The vorticity was obtained by visualizing the vortex area. In this study, the second invariant of the velocity gradient tensor (Q) was determined as the vorticity.


4 $$Q=\frac{1}{2}\left\{{\left(\frac{\partial u_{i}}{\partial x_{i}}\right)}^{2}-\frac{\partial u_{i}}{\partial x_{j}}\frac{\partial u_{j}}{\partial x_{i}}\right\}i,j=1,2,3$$

where u represents the velocity of the local water flow. Each vortex area was defined as the sum of the aggregated cells with Q > 0. Vortices around the swimmer’s body were separately determined at the ventral side of the trunk, dorsal side of the waist, and dorsal side of the shoulder, and behind the swimmer (Figure 2A). The vertical axis regions of each site were defined as between -0.75 m below and 1.0 m above from the swimmers’ body. The long axis regions for the ventral side of trunk, dorsal side of the waist and dorsal side of the shoulder were determined as based on the swimmers’ segment length. The horizontal axis region of behind the swimmer was defined as the area between the swimmer’s toes and 1.0 m behind the swimmer. The largest vortex of which the center of vorticity was located within each site of three regions except for behind the swimmer was selected for data processing (Figure 2A). All vortices generated for behind the swimmer were selected for data processing. Furthermore, when vortices were separated to sub-vortices, only the separated vortex leaving from the original vortex was selected for data processing. The original vortex was defined as the largest vortex within each site of three regions except for behind the swimmer at the start point of the downward kick. The original vortex for behind the swimmer was defined as a generated vortex which was located at the closest positions relative to the tip of the third finger. The position of the center of vorticity on the coordinate system of the simulation model of each vortex was determined around each site using the weighted average method in the sagittal plane (Figure 2A). The displacement of the center of vorticity was determined using the coordinates of vorticities at the closest and farthest positions relative to the tip of the third finger using the coordinates of vorticities of one cycle for obtaining the horizontal velocity of the center of vorticity (Figure 2B). The horizontal velocity of the center of vorticity was obtained by dividing the displacement of the center of vorticity by the displacement time (Figure 2B). The water flow velocity was subtracted from the horizontal velocity of the center of vorticity. The peak value of the vortex area was determined as the calculated area of displacement of the center of vorticity (Figure 2B). The circulation (Γ) of each vortex was obtained at the time-point of finding the peak value of the vortex area.

5 Γ=∮udr

where **u** is the velocity vector of the periphery of vortex, **dr** is the corresponding differential tangent vector. The center of vorticity, area and circulation were determined using MATLAB (2019a, Mathworks Corp.).

To verify the simulation results, the magnitude of the drag force determined in this study was compared with the corresponding values reported in previous research (Pacholak et al., 2014) (Table 1).

### Statistical analysis

All outcome data are presented as mean ± standard deviation. The normality of each data point was checked using the Shapiro-Wilk test. When the normality distribution was confirmed, the relationships between the horizontal center of mass velocity and vortex variables were assessed using Pearson’s correlation coefficients. When the normality distribution was not confirmed, the correlation coefficient was determined using the Spearman’s rank correlation coefficient. Statistical analyses were performed using IBM SPSS statistics software (Ver. 26.0, IBM Corp) and *p* < 0.05 was considered statistically significant.

## Results

**Figure 3:**
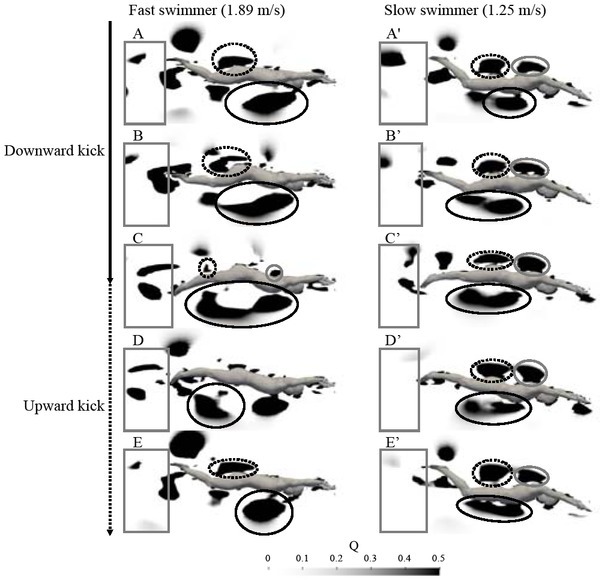
The solid black, dot black, and gray circle (or ellipse) indicate the vortices of the venal side of the trunk, waist, and shoulder, respectively. The gray rectangle indicates the vortex behind the swimmer. A(A’): the starting point of the downward kick, B(B’): during the downward kick, C(C’): the finishing point of the downward kick and starting point of the upward kick, D(D’): during the upward kick, E(E’): the finishing point of the upward kick.

The simulation results of one fast swimmer and one slow swimmer are presented in Figure 3. The horizontal velocity of the center of vorticity, the peak value of the vortex area and circulation of vortices around the swimmer’s body are presented in Table 2. No significant correlations were found between the horizontal velocity of the center of vorticity for the ventral side of the trunk, dorsal side of the waist and shoulder, and behind the swimmer, and the horizontal center of mass velocity (Table 2). The horizontal center of mass velocity was positively correlated with the peak value of the vortex area around the ventral side of the trunk, and behind the swimmer. The peak value of the vortex area around the dorsal side of the shoulder and waist was not significantly correlated with the horizontal center of mass velocity. The circulation for the ventral side of the trunk and behind the swimmer was correlated with the horizontal center of mass velocity. No significant correlations were found between the horizontal center of mass velocity and circulation for the dorsal side of the waist and shoulder.

**Table 2:** Data of horizontal velocity of the center of vorticity, the peak values of the vortex area and circulation, and the correlation coefficient between the horizontal center of mass velocity and vortex variables during UUS.

Side of the vortex	Horizontal velocity of the center of vorticity (m/s)			Peak value of the vortex area (m^2^)			Circulation (m^2^/s)		

	mean	± SD	r	mean	± SD	r	mean	± SD	r
Ventral side of the trunk	-0.50	± 1.34	-0.434	0.13	± 0.07	0.938^*^	0.69	± 0.34	0.915^*^
Waist	0.54	± 0.75	-0.433	0.07	± 0.02	0.217	-0.45	± 0.23	-0.460
Shoulder	0.31	± 0.80	0.283	0.05	± 0.03	-0.533	-0.36	± 0.21	0.350
Behind swimmer	-1.05	± 1.16	0.259	0.03	± 0.03	0.738^*^	-0.09	± 0.09	-0.680^*^

* : p < 0.05 The positive and negative value of circulation indicates clockwise and counterclockwise, respectively.

**Figure 4:**
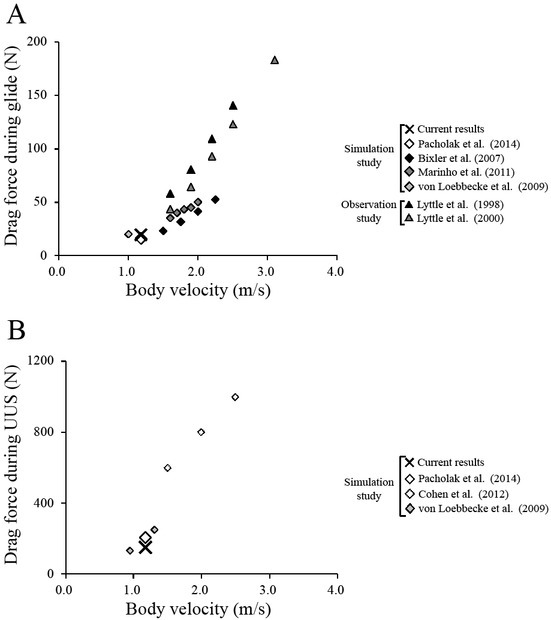
The drag force during glide **(A)** and UUS **(B)** of one swimmer of this study compared with previous studies. The cross and rhombus marks represent the current data and the simulation data of previous studies (Bixler et al., 2007; Marinho et al., 2011; Pacholak et al., 2014; von Loebbecke et al., 2009), respectively. The triangle marks represent the observation data of previous studies (Lyttle et al., 1998, 2000).

For verification of the simulation results, the peak drag force was calculated as 19.3 N and 153.4 N during the glide phase and UUS, respectively (Figure 4). The differences between the results of this study and previous research (Pacholak et al., 2014) were 3.4 N and 17.6% for the glide phase, and 54.2 N and 26.1% for UUS.

## Discussion

This is the first study to quantitatively investigate the correlation between horizontal body velocity, shedding velocity, and the area and circulation of vortices around the whole body during UUS. The main results of the current study are as follows: the vortex area and circulation around the ventral side of the trunk and behind the swimmer were positively correlated with the horizontal body velocity during UUS; and no significant correlations were found between the horizontal velocity of the center of vorticity around the ventral side of the trunk, dorsal side of the waist and shoulder, and behind the swimmer, and horizontal body velocity. Therefore, generating a large and strong vortex around the trunk and behind the swimmer may be associated with great horizontal body velocity during UUS.

The current results show that the large vortex area and circulation around the trunk and behind the swimmer may be related to achieving great horizontal body velocity during UUS. In the vortex of the ventral side of the trunk (solid black cycle), the vortex was generated from the thorax to the abdomen at the starting point of the downward kick (Figure 3A, A’), and transported and shed to the thigh during the upward kick (Figure 3B to D, B’ to D’). The vortex was observed behind the swimmer (gray rectangle) during the downward kick, and shed behind the swimmer at the finishing point of the downward kick (Figure 3A to D, A’ to D’). These vortices were generated and shed behind the swimmers’ center of mass. Previous studies suggested that marine animals generated and shed the vortex backward of their body when propel forward (Gemmell et al., 2015; Sakakibara et al., 2003; Wolfgang et al., 1999), therefore, those vortices would induce an increase in the propulsion fluid force during human UUS. Generating the larger vortex with greater circulation induces a greater changing momentum of the large volume of water, which increases the propulsion force during UUS (Drucker and Lauder, 2005; Imamura and Matsuuchi, 2013; Matsuuchi et al., 2009; Triantafyllou et al., 2002). These results indicate that generating a large and strong vortex behind the swimmer’s center of mass would contribute to achieving better UUS performance.

The horizontal velocity of the center of vorticity for each area did not correlate with the horizontal body velocity. Previous studies reported that the vortex shedding velocity or jet flow velocity was much slower than swimming velocity in fishes (Drucker and Lauder, 2005; Nauen and Lauder, 2002). Previous studies also suggested that the vortex or water shedding velocity would not contribute to increasing the body velocity in fishes (Drucker and Lauder, 2005). The current vortex shedding velocity for each area was also smaller than horizontal body velocity during UUS (Tables 1 and 2). This suggests that swimmers also may not be able to increase the vortex shedding velocity enough to contribute to produce the great body velocity using undulatory movement. Therefore, swimmers may select the strategy of generating the large and strong vortex rather than increasing the vortex shedding velocity for producing the great horizontal body velocity during UUS.

The current study evaluated the vortex structure around swimmers and investigated the horizontal velocity of the center of vorticity, the area and the circulation of vortices. The results of the current simulation study were compared with those of previous studies using CFD or particle imaging velocimetry (Cohen et al., 2012; Lyttle et al., 2000; Pacholak et al., 2014; von Loebbecke et al., 2009). The drag forces of previous and the current study were proportional to the square of the flow velocity (Figure 4). The difference in the drag forces between the current and the previous results was dependent on the physique of swimmers, the turbulence model, and/or simulation variables such as water density, dynamic viscosity, and Reynolds number. The current values of the drag force during the glide phase and UUS of one swimmer were similar to the values obtained from a previous study of the same body velocity (Pacholak et al., 2014). Moreover, the vortex structure was similar to that of previous studies that investigated the vortex structure during UUS (Cohen et al., 2012; Pacholak et al., 2014; Shimojo et al., 2019; von Loebbecke et al., 2009) (Figure 3). These results confirmed the validity of the current simulation results.

One limitation should be acknowledged when interpreting the current results. The current simulation model was set in a water flume; however, competitive swimming races are conducted in a swimming pool. Thus, the differences in the water stream around a swimmer’s body (i.e., static water or counter water flow condition) could affect the vortex structure during UUS. Nonetheless, the current results are in agreement with the results of a previous study, in which the vortex structure during UUS was simulated using CFD under static water conditions (Cohen et al., 2012). Hence, this limitation may have little effect on the main findings of this study.

## Conclusion

The current study investigated whether the horizontal vortex velocity, the area and circulation around the whole body are correlated with the horizontal body velocity during UUS. The main findings of this study were as follows: 1) the vortex area and circulation around the ventral side of the trunk and behind the swimmer were positively correlated with the horizontal body velocity, and 2) the horizontal velocity of vorticities around the whole body was not correlated with the horizontal body velocity during UUS. These results suggest that generating a large and strong vortex around the trunk and behind swimmers is related to great UUS performance.
